# MBA degree is needed for leadership roles in Medical Physics profession

**DOI:** 10.1002/acm2.12212

**Published:** 2017-11-10

**Authors:** Alonso N. Gutierrez, Per H. Halvorsen, Yi Rong

**Affiliations:** ^1^ Department of Radiation Oncology Miami Cancer Institute Miami FL USA; ^2^ Department of Radiation Oncology Lahey Hospital and Medical Center Burlington MA USA; ^3^ Department of Radiation Oncology University of California Davis Comprehensive Cancer Center Sacramento CA USA

## 1 INTRODUCTION

Medical Physics 3.0 (MP3.0) advocates that the roles of medical physics need to be redefined and reinvigorated, and furthering leadership roles was identified as a key focus. Inevitably, most medical physicists are called upon to be actively involved in major decision making at their place(s) of business, and this includes managing human resources, administrative oversight, consulting, budgeting, grand capital purchasing, and strategic planning, all of which are duties that require a wide array of leadership qualities. In an effort to address the need to improve leadership in the medical physics profession, the AAPM Summer School in 2016 provided a focused and hands‐on environment for medical physicists who had interests in developing their leadership and management skills. As an outgrowth of these activities, the AAPM has formed a Leadership Academy Working Group on providing resources and course training for medical physicists to further improve those skills. These approaches to improve leadership among our AAPM members are surely helpful, but are they sufficient? In that regard, many of our physician colleagues have adopted a different tactic, and it is common to see dual degree of M.D./M.B.A.. I herein take a notion for this debate, and we pose the question: *is an MBA degree needed for leadership roles in Medical Physics profession?* To address the question from different perspectives, Dr. Alonso N. Gutierrez argues for the proposition that “an MBA degree is needed for leadership roles in Medical Physics profession”, and Mr. Per H. Halvorsen argues against.

Dr. Alonso N. Gutierrez received his Ph.D. in Medical Physics from the University of Wisconsin‐Madison in 2007, his M.B.A. in Business of Health from the University of Texas San Antonio in 2016 and was certified by the American Board of Radiology in 2010. He is currently Chief Physicist for the Department of Radiation Oncology at the Miami Cancer Institute where he oversees both the photon and proton physics divisions. Additionally, he serves as an Associate Professor and Vice Chair of the Department of Radiation Oncology at Florida International University (FIU) Herbert Wertheim College of Medicine. Dr. Gutierrez has authored and co‐authored a number of peer‐reviewed journal articles and has been an active volunteer in professional societies serving on multiple AAPM committees, chairing the ACR TXIT physics section, and serving as an item writer for ABR.

Mr. Per H. Halvorsen received his M.S. in Radiological Medical Physics from the University of Kentucky in 1990, and was certified by the American Board of Radiology in 1995. He has practiced in large academic and community hospital settings, including a term as Vice President of Medical Physics for a company operating Radiation Oncology centers nationwide. He is currently Chief Physicist in Radiation Oncology at Lahey Health in suburban Boston. Mr. Halvorsen has been an active volunteer in professional societies, chairing the Professional Council of the AAPM and serving on the Board of Directors. He is a volunteer surveyor for the American College of Radiology, serving on its accreditation program oversight committee for many years. He is Associate Editor‐in‐Chief of the open‐access JACMP.

## 2 OPENING STATEMENTS

### 2.A Alonso N. Gutierrez, Ph.D., MBA

As the profession of medical physics has evolved over the years, the role of a medical physicist changes along with the times. The idea that a medical physicist is solely a technical expert in facilitating the calibration, measurement, and safe administration of radiation for treatment is clearly long gone. With the ever‐increasing complexities of new technology, there appears to be a growing need for team‐based treatment approaches requiring highly specialized, focused team members—above and beyond what we currently practice in most clinical settings. In this new landscape, it is my belief that the role of a medical physicist will need to flourish into one requiring medical physicists to not only provide technical guidance but also *lead* and champion the strategic implementation, clinical adoption and safe usage of novel technologies.

With this in mind, it then appears that the skill set of medical physicists needs to be further enhanced to include robust leadership and managerial training. If one reviews the current course work of many CAMPEP‐accredited, graduate medical physics training programs in the US, few, if any, mandate formal leadership and management courses as part of their degree completion requirements. In efforts to provide leadership training, some accredited medical physics residency programs include informal seminars on leadership, although the majority still do not offer formalized training. Most recently, the AAPM has brought about leadership training opportunities by focusing the 2016 AAPM Summer School topic on medical physics leadership.

Now, it is well known that there is no set recipe to ensure great leadership. In fact, the sole concept of leadership has been widely studied and to‐date, no exact definition exists. However, in attempting to define leadership, people have described common elements believed to be core to understanding successful leadership. Some of these elements have been identified as vision, motivation, creativity, thoroughness, managing ability, team building, risk tasking, and continuous improvement. Although it is known that formalized training does not guarantee successful leadership, I am a firm believer that formalized training has a role in improving the level of leadership quality within individuals.

In the context of this editorial, the topic we discuss is not whether leadership skills are needed in medical physics—as I think we can all agree that they are—but rather how medical physicists come about acquiring leadership skills. I argue that leadership is not solely nature but that medical physicists must nurture leadership skills through formalized training. In particular, I reason that the programmatic approach afforded by a Master's degree in Business Administration (MBA) enhances a medical physicist's leadership ability since MBA programs aim to teach many of those specific elements associated with successful leadership.

A common misconception with a MBA is that the degree is primarily for jobs in business or finance. I, like others, attest that the skills set taught in MBA programs is broad in nature and spans many professions. In fact, the number of MBA degrees conferred annually has seen explosive growth over the last few decades and now averages more than 150,000 annually.^1^ Specifically regarding medical physics, I argue that a MBA plays a substantial role as the responsibilities of medical physicists are broad and not just limited to technical issues. For example, it is not uncommon for medical physicists to be tasked with strategic planning of organizational growth, acquisition of capital equipment and staffing, implementation and project management of new technology, negotiation of capital purchases, organizing operational activities, leading and motivating a team, talent acquisition, or developing operating budgets. Now, one may argue that these responsibilities are associated with physicists in leadership positions; however; I dispute that responsibilities such as team building, motivating colleagues, continuous improvement, and project management are duties associated with medical physicists at all career levels. It is vital to note that possessing good leadership skills enables individuals to motivate and inspire others—this skill is valuable irrespective of whether or not one is in a management position.

Ultimately, leadership in medical physics is not an exact science and some have developed great leadership skills through unique mentorship. However, for the vast majority of physicists, I maintain that the core curriculum common to MBA programs serves to provide comprehensive, formalized training in those core elements (e.g., vision, motivation, team building, etc.) associated with successful leadership. In particular, elements such as managing and team building are taught in management and organizational behavior courses and are undoubtedly vital skills for medical physicists as they build teams to implement new technologies. Courses in strategic management teach physicists skills and tools needed to motivate and engage team members so that they may capitalize on resources to improve overall productivity. While I am not stating that *all* physicists should enroll in a MBA program, I do think it is important to recognize the need of more formalized leadership and managerial training for medical physicists as we move forward, and I firmly believe that a MBA degree is an effective pathway to develop leadership skills.

### 2.B Per Halvorsen, MS, FACR, FAAPM

The medical physics practice environment is changing. In 2014, I wrote a JACMP Editorial titled “The next decade for clinical medical physics”.^2^ The article summarized the external factors which, in my view, will force our profession to adapt. Borrowing from the article: “If we're not willing to transform the practice of clinical medical physics over the next decade, our services may become commoditized and our profession marginalized. A successful transformation for the clinical medical physics profession requires that the Qualified Medical Physicists (QMP) become a more visible consultant and resource to health systems in safety assessments and quality control (QC) program design, as well as becoming a competent manager of other technical employees, rather than limiting our scope to the familiar range of QC tests and clinical procedure support.” The same could also be said for academic medical physics.

Medical physics leaders, then, must possess additional skills in the future. In addition to the core attributes we have always expected of such leaders, the future skill set should include competency in healthcare finance, organizational leadership, and the ‘soft skills’ of motivating teams of professionals of diverse technical backgrounds.

Given the aforementioned, is a MBA degree a requirement for success as a medical physics leader in the future? I posit that it is a valuable supplement, but not essential.

The MBA programs can provide a different and useful perspective, and perhaps Healthcare MBA programs are particularly well suited in this regard. But there are many other paths to acquiring the skills mentioned above. Active participation in leadership training opportunities coupled with active participation in professional activities within our employer institutions and/or professional societies—learning and practicing these skills—can be another productive path. The AAPM recently launched the Medical Physics Leadership Academy with sessions tailored to medical physicists,^3^ and the American College of Radiology (ACR) Leadership Institute provides similar programs.^4^ Many large employer organizations provide leadership and business training courses. To practice what we have learned, volunteering for institutional initiatives that are multidisciplinary can be an effective way to wet one's toes. For example, contributing to an institution‐wide safety initiative requires good data analysis, process analysis, and staff education/training, all of which are core elements in leadership quality.

A quick look at the list of past AAPM Presidents provides some historical precedents of outstanding leaders who acquired their skills through other paths. I have been privileged to know some of them well. My early career mentor Bengt Bjarngard was an exceptional medical physicist who embraced the importance of multidisciplinary collaboration and became a driving force in building one of the most respected radiation oncology programs in the world. More recently, Mike Herman guided the profession through a significant challenge with the mainstream‐media coverage of serious adverse events and subsequent Congressional hearings, and guided the collaboration with our physician sister society in addressing the safety concerns.

But what if historical precedent is not relevant to the new and evolving practice environment? Exceptional “non‐MBA” contemporary leaders such as Jessica Clements continue to show how a clinical medical physicist can be a visible and respected professional leader in their institution while collaborating with physician‐led societies to prepare the professionals of tomorrow.

Medical physicists are well positioned to be professional leaders in the healthcare setting—we have a strong foundation in science, good data‐analytics skills, and a keen understanding of how to apply this toward good patient care. Our core skill set should allow us to gain a basic competence in healthcare finance if we invest the focus and effort. The crucial “soft skills” needed for success in the years ahead can be acquired through many paths—MBA programs are a useful complement in this regard, but they are not an essential ingredient.

## 3 REBUTTAL

### 3.A Alonso N. Gutierrez, Ph.D., MBA

First, I want to commend Per for highlighting a crucial statement he made in his JACMP Editorial in 2014 where he stated that medical physicists need to “become a more visible consultant and resource to health systems in safety assessments and QC program design, as well as becoming a competent manager of other technical employees.” While I do whole‐heartedly agree that safety assessment and QC program design are important for our profession to bring new value to our healthcare organizations, I believe that these skills are ones that medical physicists are intrinsically capable of learning, for they are skills that tend to be logistical and analytical in nature.

What I feel will be a tougher challenge for medical physicists, simply because it is not commonly taught in their dense math and physics training curricula, is the ability to be “competent in healthcare finance, organizational leadership, and the ‘soft skills’ of motivating teams of professionals of diverse technical backgrounds.” It is this particular skill set I know firsthand is rigorously taught, both didactically and practically, in the core courses of any given MBA program. While it is true that a minority of medical physicists may naturally possess these skills, I again feel that a significant majority of medical physicists can obtain/enhance these skills through the formalized, programmatic approach of a MBA degree.

As Per mentions, active participation in leadership training opportunities coupled with active participation in professional activities may potentially serve as an avenue to develop these skills. I, however, caution that many of these leadership training opportunities are somewhat limited in applicability as they tend to be “blitz” training sessions with numerous leadership and management concepts thrown at attendees over the course of a few hours or days. In this short time period, it is hard to fully digest and fundamentally comprehend key concepts that are inherent to becoming an effective leader—take, for example, the notion of team building. While a short leadership session might provide some “tips or tricks” as to how to better build a team. A MBA degree will not only teach those tools but will also impart deeper knowledge as to how to effectively motivate that team (such as applying various motivational models, for example, Maslow's Hierarchy of Needs, Alderf's ERG Theory, or the Expectancy Theory) and promote true communication within the team (by utilizing various communication channels and levels of communication richness).

While I myself have also been privileged to work alongside medical physicists with outstanding leadership ability, I genuinely feel that luck was on my side and that my on‐the‐job mentorship experience was one not common to all. Having been privy to both a MBA and wonderful leadership mentorship, I can attest that the depth of understanding of leadership, finance and management concepts through my MBA studies is hard to be reproduced elsewhere. With this in mind, I cannot help but echo Per's statement that “Medical physicists are well positioned to be professional leaders in the healthcare setting.” I firmly believe this to be the case, and I strongly advocate that CAMPEP accredited graduate and residency programs move to provide formalized leadership and management courses to students and trainees. Similarly, for the practicing medical physicists, I encourage you to seek out programmatic leadership training opportunities, be it a few courses or a complete degree, as it will serve to positively compliment your current skill set.

### 3.B Per Halvorsen, MS, FACR, FAAPM

Dr. Gutierrez and I agree that medical physicists must be able to lead teams of professionals in the safe implementation of modern medical technology—and we agree that current medical physics graduate and residency programs do not sufficiently address the skills needed in this regard.

As Dr. Gutierrez stated, MBA programs provide structured training in essential topics such as organizational behavior, management and team building. These are the very same topics covered by other professional leadership programs such as those mentioned in my opening statement. Of course, a program lasting days or weeks cannot explore these topics in the same depth as a semester‐long course devoted to a single topic. Many business schools provide certificate courses for active professionals, and this can be an excellent venue for acquiring the necessary knowledge. To build upon that knowledge, one must practice what was learned—and this is no different for an individual who pursues the MBA path.

Medical physics graduate and residency programs can play an important role as well, by building awareness of the importance of these skills for a successful career in medical physics. If new entrants into our profession consider these skills as equally important, and include them in their lifelong learning objectives, the profession will be well served. The MBA can be a valuable complement in this regard, but it is not essential.

## CONFLICT OF INTEREST

No conflicts of interests for all authors.
